# Dacryocystitis secondary to neglected silicone tube in lacrimal duct for 10 years: A case report

**DOI:** 10.1097/MD.0000000000023073

**Published:** 2020-11-06

**Authors:** Lanjian Li, Zhaoguang Lai, Wei Huang, Fan Xu, Yu Wu

**Affiliations:** a Department of Ophthalmology, People's Hospital of Guangxi Zhuang Autonomous Region; b Guangxi Medical University, Nanning 530021, Guangxi, People's Republic of China.

**Keywords:** case report, complications, contracture, dacryocyst, silicone tube

## Abstract

**Introduction:**

Contracture of dacryocyst by an implanted lacrimal silicone tube is rare. This report describes a unique case of secondary dacryocystitis and the contracture of dacryocyst caused by a lacrimal silicone tube that was placed in the lacrimal system for 10 years.

**Patient concerns:**

A 63-year-old female was diagnosed with chronic dacryocystitis at a local hospital and underwent surgical treatment 10 years ago. In the past month, the patient complained of persistent tearing and purulent secretion from the eyes.

**Diagnosis:**

The patient was diagnosed with secondary dacryocystitis, based on clinical features and the presence of the silicone stent, granulation tissue formation, and dacryocyst contracture in the lacrimal duct, as observed by nasal endoscopy.

**Interventions:**

For treatment, the implanted silicone tube in the patient was removed, the lacrimal duct and nasal mucosa was anastomosed, and a new lacrimal silicone tube was placed again.

**Outcomes:**

Following the surgery, the patient recounted that there were no symptoms, and follow-up examinations performed over a 1-month period posttreatment revealed no recurrence of obstruction or dacryocystitis. Therefore, the surgeon removed the lacrimal drainage tube and asked the patient to return to the outpatient department regularly for examination.

**Conclusion:**

The findings, in this case, suggest that silicone tubes are safe and effective, and can be placed in the lacrimal drainage system. However, in this patient, prolonged intubation caused chronic inflammation, granulation tissue formation, and dacryocyst contracture. Our findings could inform surgeons to consider the reasonable duration of intubation for treating cases of lacrimal obstruction, in order to avoid unnecessary complications.

## 1 Introduction

Tearing, blurred vision, ocular and facial pains, and recurrent conjunctival discharge are common clinical manifestations and signs of lacrimal duct obstruction.^[1–3]^ These symptoms, which are ascribed to lacrimal duct obstruction, frequently present in middle-aged and elderly patients. Chronic dacryocystitis (CD) is generally attributed to the effects of obstruction or stenosis of the lacrimal outflow system, which can be due to neoplasms, traumatic injuries, or inflammation due to unknown causes.^[3]^ The tears and secretions which stagnate in the lacrimal sac could easily trigger bacterial infections.^[4]^ Although the symptoms of the disease may be relieved by conservative treatment in the early stages, CD is rarely cured entirely. Thus surgical treatment needs to be considered at this stage. Silicone tube intubation of the lacrimal outflow system plays a critical role in the treatment of lacrimal duct obstruction in CD

The lacrimal silicone tube should be placed in situ and should be kept maintaining patency for a sufficient amount of time. This helps the damaged mucosa become re-epithelialized and avoids relapses in the lacrimal duct obstruction. But the infection of these devices can lead to chronic conjunctivitis, dacryocystitis, scarring of the lacrimal system, and recurrent epiphora.^[5]^ However, there are no standard clinical recommendations for the amount of time the silicone tube can remain implanted up until now. According to previous research, the amount of time that the silicone tube has been placed in the lacrimal duct ranges from 1 week to several years, and a timeframe of 2 to 6 months has been most frequently cited in the past 2 decades.^[6–10]^

We report a case on secondary dacryocystitis and contracture of dacryocyst due to a lacrimal silicone tube that had been placed in the lacrimal system for 10 years. In this case, we researched the condition of the lacrimal sac and mucosa using nasal endoscopy. In addition, we evaluated the effect of surgery after tube removal.

## 2 Case presentation

**Figure 1:**
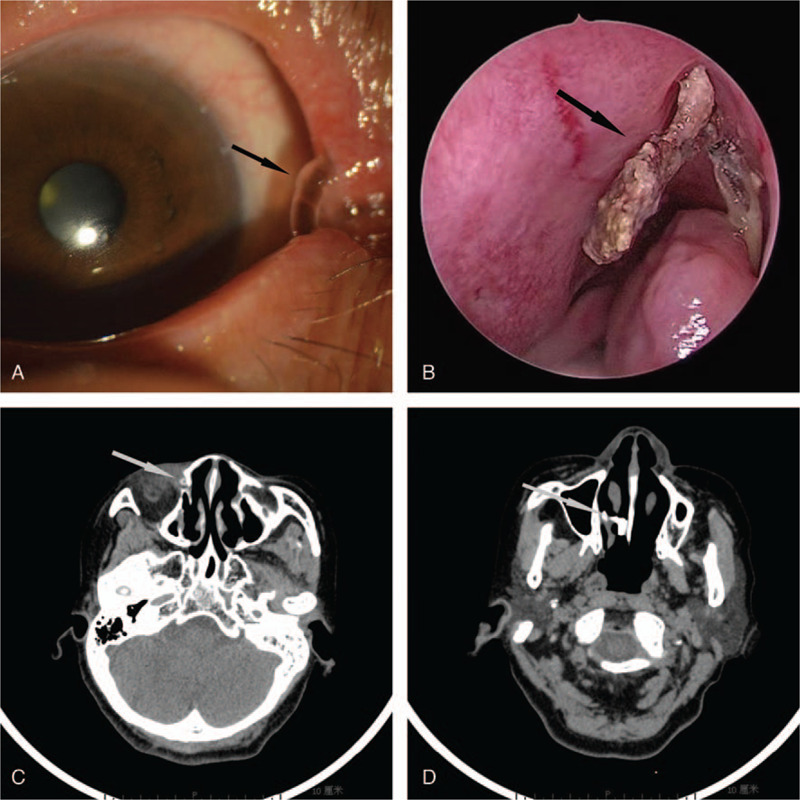
Clinical photographs of the puncta (A), including a view of the inferior nasal meatus using nasal endoscopy (B) and CT scan (C, D). The low density in the center of the silicone tube indicates that the tube is unobstructed (C), but the high density calcification can be seen at the end (B, D). Proper insertion of a silicone tube is shown (arrows). CT = computed tomography.

A 63-year-old female complained about a 1-month history of epiphora and discharge in her right eye. She had a past medical history of CD, and had been treated by reconstructive surgery for silicone stent intubation on her right lacrimal duct at a local hospital 10 years earlier. But, it is noteworthy that the patient had not returned to the outpatient department of ophthalmology for a follow-up examination after the surgery. When the patient initially came to our outpatient department for treatment, we found the silicone stent in the appropriate position by slit-lamp microscope examination, computed tomography (CT), and nasal endoscopy (Fig. 1 A,B). The low density in the center of the silicone tube indicated that the tube is unobstructed, but high-density calcification can be seen at the ends (Fig. 1B-D).

**Figure 2:**
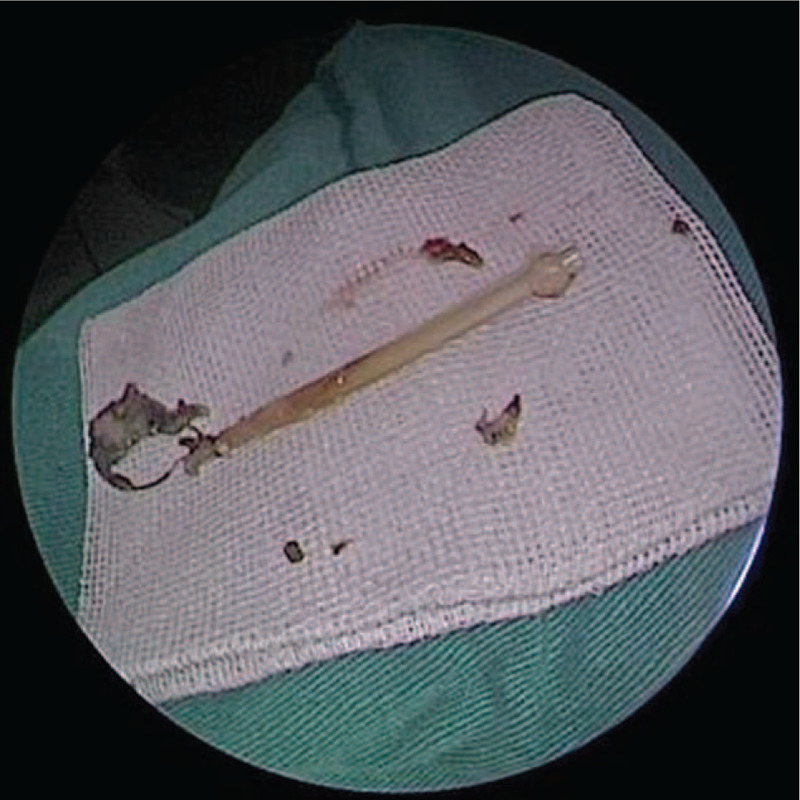
Detailed photographs of the removed lacrimal tube. Accumulation of pus was seen in the inner cavity of the tube.

Although we did not find evident lesions of the lacrimal drainage system due to the previous surgery, dacryocystitis accompanied by secretion formation was seen, located around the lacrimal passage. We observed that the lacrimal passage was obstructed, because when the irrigation solution was irrigated through the lower punctum, the solution created a reflux from the upper punctum, and the patient could not swallow the solution through the throat. Soft tissue density, which was unclear in boundaries, was seen at the lacrimal sac and nasolacrimal duct level on the dacryocystography. We concluded that there was inflammation at the lacrimal sac and nasolacrimal duct level. Thus, a surgical therapeutic schedule was followed. We irrigated the lacrimal duct and instilled antibiotic eye drops for the patient. After the inflammation had settled, the tube removal was performed by an experienced surgeon. During the operation, granulation tissue formation and dacryocyst contracture, which makes the lacrimal sac lose function due to secondary dacryocystitis in the lacrimal drainage system, was observed by nasal endoscopy. Therefore, the surgeon performed an operation that anastomosed the lacrimal duct and nasal mucosa, and placed a lacrimal silicone tube again. The removed tube was identified as a lacrimal silicone stent (BLZ-Fr8; Freda Corporation, Shandong, China). Accumulation of pus was seen in the inner cavity of the tube (Fig. 2). The silicone tube that was implanted was the lacrimal drainage tube (Fr3; Freda Corporation, Shandong, China).

Following the surgery, the patient recounted that there were no symptoms and discomfort, and follow-up examinations performed over a period of 1 month posttreatment revealed no recurrence of obstruction or dacryocystitis. Thereafter, the surgeon removed the lacrimal drainage tube and asked the patient to return to the outpatient department regularly for examination.

## 3 Discussion

CD refers to chronic catarrhal or suppurative inflammation of the lacrimal sac. It is commonly attributed to the effects of obstruction or stenosis of the lacrimal outflow system. The most common reason for CD is the nasolacrimal duct obstruction (NLDO), which can be due to neoplasms, traumatic injuries, or inflammation due to unknown causes.^[11]^ These situations could cause the tears to stagnate in the lacrimal sac and stimulate the mucous membrane of the lacrimal sac for a long time, triggering repeated bacterial infections. Drugs can control the early stages of the disease, but the condition needs timely surgical treatment in the later stages.

External dacryocystorhinostomy (DCR) is deemed as the standard surgical treatment for the NLDO. This method of treatment can help visualize the anatomical structures of the lacrimal sac, but will also lead to a scar beside the medial canthal ligament and cause potential damage. The effects of endoscopic dacryocystorhinostomy (eDCR) are not only equivalent to the external approach, but also have distinct cosmetic advantages.^[12–14]^ However, in either case, the two procedures are invasive. Therefore, reconstructive surgery by silicone tube intubation has been employed in some cases of epiphora.

Since the early 1900s, people have used alloplastic materials to augment the soft tissue. Since being introduced to medicine in the early 1950s, silicone rubber products have supplanted most other alloplastic materials where soft implants are indicated. The acceptance of the silicone has been mainly due to their low tissue reactivity and successful clinical applications.^[15,16]^

The narrow soft tissue of the lacrimal drainage system can be dilated by the placement of a silicone stent, thus allowing increased flow of tears. In a study by Moscato et al, the median duration of silicone intubation is 5.7 years by Kaplan-Meier survival analysis.^[17]^

Masashi et al suggested that the lacrimal system has to undergo re-epithelialization after surgery. Lacrimal intubations can therefore be left in situ for the long-term, since the silicone tube is inert.^[18]^

The reason why some studies have indicated that the silicone tube could remain safely in situ indefinitely is because a study by Angela et al found a zero complication rate for patients with tubes in situ for more than 36 months. They concluded that the silicone nasolacrimal tube is inert, so the tube should be left in situ as long as the patient can tolerate the tube well, and there is no residual epiphora. This approach can reduce or circumvent the potential risk of damaging the lacrimal system while removing the silicone tube.^[19]^

Silicone tube intubation produces a marked effect in the treatment of lacrimal duct obstruction. It should be noted that a controversy exists regarding how long the lacrimal tube should be left in situ before removal. But, there are few former reports describing the periods for which the silicone tube can be left in the lacrimal drainage system.

However, a study by Moscato et al mentioned that the success rate of silicone tube intubation was 96% at 2 years, but dropped down to 85% at 3 years. Moscato estimated that the success rate of silicone intubation would be reduced to 50% after 5 years.^[17]^

Also, Aakalu et al proposed that the median time of Jones tube extrusion is 8 months and the mean time is 16 months.^[7]^ In Minwook's report, the success rate of lacrimal duct intubation was close to 88% at 6 months, but there was a reduction of more than 20% at 2 years. That means the longer placement of silicone tube lowers the success rate in treatment.^[8]^

Ben Limbu's analysis has demonstrated that the success rate of silicone tube removal 2 weeks after operation was 93.5%, and that this approach can relieve the financial burden placed on patients. This result is not markedly different from the outcome or complication rates of silicone tube removal after 6 weeks, reported in other countries.^[9]^

Our focus in this report was to determine whether there are complications after taking out the silicone tube. David B. Samimi's study demonstrated that all of the patients with noninfected stents had not exhibited residual epiphora, in comparison to 22% of the elimination rate of epiphora in the patients with infected stents.^[10]^

It is thus clear that intubation devices that are implanted during lacrimal surgery can lead to chronic inflammation, early device migration, deformation of the lacrimal tube, contractures of the dacryocyst, and scarring of the lacrimal system, causing recurrent epiphora.^[5,20–22]^ These studies suggest that the lacrimal silicone tube should be taken out early after lacrimal intubations, on the basis of ensuring its therapeutic effects.

In our present case, the female patient had a past medical history of CD, and had been treated by reconstructive surgery for silicone stent intubation on her right lacrimal duct at a local hospital 10 years ago. She had not returned to the outpatient department of ophthalmology for a follow-up examination after the surgery. When the patient initially came to our outpatient department for treatment, CD was found around the lacrimal passage, accompanied by secretion formation in the nasolacrimal duct. We began a surgical treatment regimen within 3 days, after the inflammation was settled. During the surgery, granulation tissue formation and dacryocyst contracture, which let the lacrimal sac lose function due to secondary dacryocystitis in the lacrimal drainage system, was found by nasal endoscopy. So, the surgeon performed an operation that anastomosed the lacrimal duct and nasal mucosa, and placed a lacrimal silicone tube again.

Following the surgery, the patient recounted that there were no symptoms, and follow-up examinations performed over a month posttreatment revealed no recurrence of obstruction or dacryocystitis. Therefore, the surgeon removed the lacrimal drainage tube and asked the patient to return to the outpatient department regularly for examination.

## 4 Conclusion

The findings in the current case suggest that the secondary dacryocystitis in our patient resulted from a reaction to the retained lacrimal stent for a long time. Our findings reveal that the silicone lacrimal tube should be removed after lacrimal intubations early on, even though silicone tubes have low tissue reactivity. If the lacrimal tube is left in situ long-term, it can cause chronic inflammation, deformation of the lacrimal tube, contractures of the dacryocyst and scarring of the lacrimal system.

## Acknowledgments

The authors would like to thank the patient for their participation in this study. Thanks for Fan Xu and Yu Wu's patient guidance. Thanks for Huang Wei's cooperation.

## Author contributions

**Conceptualization:** Lanjian Li, Zhaoguang Lai, Fan Xu, Yu Wu.

**Data curation:** Lanjian Li, Zhaoguang Lai, Fan Xu, Yu Wu.

**Formal analysis:** Lanjian Li.

**Investigation:** Lanjian Li, Zhaoguang Lai, Fan Xu, Yu Wu.

**Methodology:** Lanjian Li, Yu Wu.

**Project administration:** Yu Wu, Fan Xu.

**Resources:** Lanjian Li, Zhaoguang Lai, Fan Xu, Yu Wu.

**Supervision:** Lanjian Li, Yu Wu, Fan Xu.

**Validation:** Lanjian Li, Zhaoguang Lai, Fan Xu, Yu Wu.

**Visualization:** Lanjian Li, Zhaoguang Lai, Fan Xu, Yu Wu.

**Writing – original draft:** Lanjian Li, Wei Huang, Fan Xu.

**Writing – review & editing:** Lanjian Li, Fan Xu, Yu Wu.
